# Incidence and warning signs for complications of human brucellosis: a multi-center observational study from China

**DOI:** 10.1186/s40249-024-01186-4

**Published:** 2024-02-20

**Authors:** Qing-Nan Shi, Hui-Jie Qin, Qiao-Shan Lu, Shu Li, Zhong-Fa Tao, Meng-Guang Fan, Mu-Heta Aishan, Zeng-Qiang Kou, Qiu-Lan Chen, Wen-Wu Yin, Yan-Ping Zhang

**Affiliations:** 1 National Key Laboratory of Intelligent Tracking and Forecasting for Infectious Diseases, Chinese Center for Disease Control and Prevention, Changbai Road, Changping District, Beijing, 102206 China; 2 School of Public Health, Guangxi Medical University, Nanning, China; 3 Nanning Center for Disease Control and Prevention, Nanning, China; 4 Guizhou Center for Disease Control and Prevention, Guiyang, China; 5 Inner Mongolia Center for Disease Control and Prevention, Hohhot, China; 6 Xinjiang Uighur Autonomous Region Center for Disease Control and Prevention, Urumqi, China; 7 Shandong Center for Disease Control and Prevention, Jinan, China

**Keywords:** Brucellosis, Complications, Clinical spectrum, Warning signs

## Abstract

**Background:**

Brucellosis is a severe zoonotic disease that is often overlooked, particularly in impoverished countries. Timely identification of focal complications in brucellosis is crucial for improving treatment outcomes. However, there is currently a lack of established indicators or biomarkers for diagnosing these complications. Therefore, this study aimed to investigate potential warning signs of focal complications in human brucellosis, with the goal of providing practical parameters for clinicians to aid in the diagnosis and management of patients.

**Methods:**

A multi-center cross-sectional study was conducted in China from December 2019 to August 2021. The study aimed to investigate the clinical characteristics and complications of patients with brucellosis using a questionnaire survey and medical record system. The presence of warning signs for complications was assessed using univariate and multivariate logistic regression models. Receiver operating characteristic (ROC) curves and the area under the curve (AUC) were used for variable screening and model evaluation.

**Results:**

A total of 880 participants diagnosed with human brucellosis were enrolled. The median age of the patients was 50 years [interquartile range (*IQR*): 41.5–58.0], and 54.8% had complications. The most common organ system affected by complications was the osteoarticular system (43.1%), with peripheral arthritis (30.0%), spondylitis (16.6%), paravertebral abscess (5.0%), and sacroiliitis (2.7%) being the most prevalent. Complications in other organ systems included the genitourinary system (4.7%), respiratory system (4.7%), and hematologic system (4.6%). Several factors were found to be associated with focal brucellosis. These factors included a long delay in diagnosis [odds ratio (*OR*) = 3.963, 95% confidence interval (*CI*) 1.906–8.238 for > 90 days], the presence of underlying disease (*OR* = 1.675, 95% *CI* 1.176–2.384), arthralgia (*OR* = 3.197, 95% *CI* 1.986–5.148), eye bulging pain (*OR* = 3.482, 95% *CI* 1.349–8.988), C-reactive protein (CRP) > 10 mg/L (*OR* = 1.910, 95% *CI* 1.310–2.784) and erythrocyte sedimentation rate (ESR) elevation (*OR* = 1.663, 95% *CI* 1.145–2.415). The optimal cutoff value in ROC analysis was > 5.4 mg/L for CRP (sensitivity 73.4% and specificity 51.9%) and > 25 mm/h for ESR (sensitivity 47.9% and specificity 71.1%).

**Conclusions:**

More than 50% of patients with brucellosis experienced complications. Factors such as diagnostic delay, underlying disease, arthralgia, eye pain, and elevated levels of CRP and ESR were identified as significant markers for the development of complications. Therefore, patients presenting with these conditions should be closely monitored for potential complications, regardless of their culture results and standard tube agglutination test titers.

**Supplementary Information:**

The online version contains supplementary material available at 10.1186/s40249-024-01186-4.

## Background

Brucellosis is a zoonotic disease that has significant negative impacts on both livestock productivity and human health worldwide, particularly in developing countries [1]. It is a disease of poverty [2], with an estimated 3.5 billion individuals at risk of contracting brucellosis globally [3]. In recent years, there has been a sharp increase in human brucellosis incidence, posing a significant public health threat in China [4]. Transmission primarily occurs through direct contact with infected animal fluids and secretions, consumption of unpasteurized or raw dairy products, or inhalation of infectious aerosols [5]. Brucellosis can affect various organs and systems, sometimes presenting with acute systemic symptoms, but may also manifest without such symptoms. Early and accurate diagnosis is crucial for effective management and treatment of brucellosis.

Brucellosis is caused by Gram-negative bacteria of the genus *Brucella*, which have the ability to establish long-term infections in their hosts [6, 7]. *Brucella* evades the host immune system’s clearance mechanisms, and the duration of infection within host cells depends on the host's immunity and the appropriate use of specific antibiotics. Therefore, early diagnosis and proper treatment are crucial in reducing the risk of chronicity and relapse. However, the clinical manifestations of brucellosis can vary greatly and mimic other diseases, leading to misdiagnosis and missed diagnosis [8]. Confirming brucellosis requires a combination of exposure history, clinical manifestations, and specific laboratory tests. Underdiagnosis of brucellosis leads to higher rates of complicated cases [9]. Focal involvement in brucellosis requires longer treatment duration and additional antimicrobial agents compared to uncomplicated cases [10]. Unfortunately, there are currently no specific biomarkers reported for diagnosing complications. It is challenging to distinguish complications based solely on general symptoms, signs, or single biomarkers [4]. Therefore, it is necessary to explore comprehensive diagnostic warning indicators for complicated brucellosis to aid clinicians in promptly diagnosing and treating the disease.

Understanding the true clinical spectrum of brucellosis and its complications is crucial for medical practitioners to make early decisions. Although a few studies have reported on the clinical spectrum of brucellosis complications and related factors [8, 11, 12], these studies had limitations such as being single center, having small sample sizes, and focusing only on inpatients, which will likely overestimate the reported complication rates, and latent indicators for identifying focal involvement in outpatients might have been missed. To address these limitations, we conducted a multi-center observational study to determine the real incidence and clinical spectrum of brucellosis complications in both inpatients and outpatients and to identify clinical and laboratory warning indicators for complications, which could improve the diagnostic criteria and enhance the management of patients. Furthermore, it is worth noting that the current version of the guideline on the diagnosis and therapy of brucellosis in China does not include diagnostic principles for complications [13]. Therefore, our study is valuable in standardizing the diagnosis and treatment of brucellosis complications, ultimately reducing the incidence of chronic brucellosis.

## Methods

### Study design and setting

A multi-center cross-sectional study was conducted in five cities in China, namely Bayannur City in Inner Mongolia Autonomous Region, Ili and Changji City in Xinjiang Uygur Autonomous Region, and Jinan and Dongying City in Shandong Province, from December 2019 to August 2021. These cities were selected based on their high incidence rates of brucellosis, being among the top 10% in their respective provincial-level administrative divisions (PLADs). All project sites were designated medical institutions for brucellosis treatment and had the necessary capacity to conduct the required examinations and laboratory tests. To maintain biosafety, *Brucella* blood cultures were performed at CDC laboratory, while other tests were carried out at the project sites. The laboratory test results were consistent across all project sites. The study protocol was approved by the Research Ethics Review Committee of the Chinese Center for Disease Control and Prevention (Approval number: 201942), and signed informed consent was obtained from all participants prior to the investigation.

### Sample size

A cross-sectional study was conducted to estimate the sample size for assessing the prevalence of focal complications in human brucellosis. The “Confidence Intervals for One Proportion” module from Power Analysis and Sample Size Software (Version 15, NCSS LLC., East Kaysville, Utah, United States) was used to perform the sample size calculation. Based on a previous study reporting a range of brucellosis complication rates from 27.7–89.7% [11, 14], we chose a conservative estimate of 50% to ensure the maximum sample size. Considering confidence levels: 95% confidence intervals (*CIs*) formula was Exact (Clopper-pearson), the width of the *CIs* (two sided) was 10%, the dropout rate was 20%, the minimum sample size was 503 individuals. Ultimately, a total of 880 brucellosis patients were included in the study.

### Sampling and participants

A multi-stage random sampling method was utilized to select specific hospitals and patients with human brucellosis. In regions of Inner Mongolia, Xinjiang, and Shandong with high incidence rates, five county-level designated medical institutions were chosen. From December 2019 to August 2021, doctors from these designated medical institutions enrolled eligible brucellosis patients who met the criteria, following the principle of informed consent. Enrollment continued until the desired sample size was achieved.

The inclusion criteria for cases were as follows: (1) patients diagnosed with brucellosis; (2) aged 15 years and above; (3) provided informed consent and willingly participated in the study.

The criteria for case exclusion were: (1) infected with the human immunodeficiency virus (HIV), undergoing chemotherapy, having other immune system disorders, or suffering from other severe illnesses; (2) suffering from mental disorders, deafness and other disorders resulting in poor communication and inability to cooperate with the investigation; (3) pregnant woman.

The definition of confirmed cases of brucellosis includes patients who have both a history of exposure and clinical symptoms, and meet one of the following criteria: (1) Isolation and culture of *Brucella* bacteria from the patient's blood or other specimens; (2) A serum agglutination test (SAT) titer of ≥ 1:100 (++) or a duration of illness of over one year with a titer of ≥ 1:50 (++); (3) A Coombs test titer of ≥ 1:400 (++).

### Data collection and definitions

After obtaining informed consent, each patient diagnosed with brucellosis was interviewed by a qualified physician using a standardized questionnaire. The purpose of the questionnaire was to gather the following information: gender, age, nationality, occupation, region of residence, level of education, exposure to animals (specifically sheep & goats, cattle, etc.), methods of animal exposure (such as raising, grazing, slaughter, delivering lambs, etc.), consumption of unpasteurized food, family history of brucellosis, and number of days since symptoms onset. Additionally, relevant variables from the Hospital Information System were extracted, such as date of diagnosis, presence of underlying diseases, previous history of brucellosis, symptoms and signs experienced (such as fever, chills, fatigue, etc.), complications, and laboratory test results.

A standardized protocol was implemented to ensure consistency in interviewer training and quality control supervision throughout all survey instances. Cases were included based on specific inclusion and exclusion criteria. Face-to-face interviews were conducted with all eligible cases by physicians who received professional training. Each questionnaire underwent thorough review by qualified supervisory staff. Data management specialists checked the collected questionnaires for completeness and logical consistency. The laboratory tests were carried out by properly trained laboratory technicians following national standardized methods and procedures.

The clinical stages of brucellosis were classified as follows: the acute stage, which lasted less than 3 months from the onset of symptoms to admission; the subacute stage, which lasted from 3 to 6 months; and the chronic stage, which lasted longer than 6 months. Age was calculated by determining the time period between the date of study participation and the date of birth for each brucellosis case. Fever was defined as axillary temperature of > 37.3 °C. Anemia: hemoglobin (Hb) female and children < 110 g/L, male < 120 g/L. Leukopenia: white blood cells (WBC) < 4 × 10^9^ /L; Leukocytosis: WBC > 10 × 10^9^ /L; Thrombocytopenia: platelet < 100 × 10^9^ /L. ESR elevation: female > 20 mm/h and male > 15 mm/h.

In this study, we defined “focal complication” as a patient with symptoms of brucellosis who has at least one affected organ and tests positive for brucellosis through serology or culture. We defined “osteoarticular involvement” as the presence of inflammatory signs (swelling, pain, functional disability, heat, or redness) in any peripheral osteoarticular location, along with radiographic evidence of abnormalities. Peripheral arthritis was diagnosed based on clinical findings of joint swelling, effusion, and limited motion, as well as X-ray imaging. Sacroiliitis, spondylitis, and paravertebral abscesses were diagnosed based on clinical findings, as well as bone scans or magnetic resonance imaging (MRI). Neurological complications were confirmed in cases where the patient had a positive *Brucella* culture and/or positive blood or spinal *Brucella* culture with abnormal cerebrospinal fluid, as well as symptoms and signs of encephalitis or meningitis, while excluding other neurological diseases. Cardiovascular complications were identified by the presence of signs and symptoms such as heart murmur, retrosternal pain, and abnormalities in electrocardiogram (ECG) or ultrasonic cardiogram (UCG), after ruling out other causes and/or with positive *Brucella* culture in cases of pericardial effusion. Hematological complications were diagnosed based on abnormal clinical manifestations (anemia or bleeding) and abnormal laboratory findings, while excluding other causes. Genitourinary complications such as Orchitis, epididymitis, and pelvic inflammation were diagnosed based on signs and symptoms of urogenital system inflammation (orchialgia, testicular enlargement, and lower back pain in men; lower abdominal pain in women), confirmed by ultrasound. Respiratory system complications presented as bronchitis, pneumonia, or pleural effusion, and could be confirmed through chest X-rays, computed tomography (CT) scans, or MRI scans once other possible causes were ruled out. Cutaneous complications were defined as clinical manifestations of skin rash, purpura, ecchymosis, erythema nodosum, ulceration, or abscess in confirmed cases of brucellosis, while excluding other causes.

### Statistical analysis

Data storage was done using EpiData version 3.1 (EpiData Association, Odense, Denmark). The normality assumption for quantitative variables was assessed using the Kolmogorov–Smirnov (KS) test with Lilliefors correction for significance. Non-normally distributed variables were described using median and inter-quartile ranges (IQRs). Categorical variables were described using frequency and percentage. Differences in proportions were tested using Pearson’s *χ*^2^ test or Fisher’s exact test. Logistic regression models were used to investigate the association between brucellosis complications and demographic, clinical, and laboratory characteristics. Univariate logistic regression was used to screen variables, considering *P* < 0.1 as statistically significant. Correlation analyses were performed using Spearman’s or Pearson’s correlation, and variables with strong correlations were removed. Factors selected from the screening were included in a multivariate analysis using a backward stepwise procedure. Multiple categorical variables were included in the model as dummy variables. Odds ratio (*OR*) and 95% *CIs* were calculated for categorical variables using a two-tailed test. Model evaluation and refinement were conducted using covariance checking, overdispersion correction, and other methods. Receiver operating characteristic (ROC) curves were generated to determine optimal cutoff values for diagnosing complicated brucellosis. Sensitivity and specificity were computed for each parameter in distinguishing complicated from uncomplicated brucellosis, and the area under the curve (AUC) was calculated. Data analysis was performed using SAS version 9.4 (SAS Institute Inc., Cary, USA). All tests were two-sided with significance set at *P* ≤ 0.05.

## Results

In our study, we enrolled a total of 880 participants diagnosed with human brucellosis. The study period spanned from December 2019 to August 2021. Among the participants, 482 individuals (54.8%) presented with complicated brucellosis. Complications were observed in seven different anatomical systems, with osteoarthritis being the most prevalent focal complication. Please refer to Table 1 for further details.

**Table 1:** Distribution of complications in 880 brucellosis cases

Complications	*n* (%)
Osteoarticular system	379 (43.1)
Sacroiliitis	24 (2.7)
Spondylitis	146 (16.6)
Peripheral arthritis	264 (30.0)
Paravertebral abscess	44 (5.0)
Genitourinary system	41 (4.7)
Respiratory system	41 (4.7)
Hematologic system	40 (4.6)
Cardiovascular system	11 (1.3)
Neurological system	9 (1.0)
Cutaneous system	1 (0.1)

### Demographic and epidemiological characteristics

Demographic and epidemiological characteristics, stratified by complicated, are displayed in Table 2. A total of 642 (80.0%) patients were male. The median age was 50 years (*IQR*: 42–58, ranged from 16 to 73 years) and about four-fifths were aged from 31 to 60 years. The proportion of complications is higher in the age group over 60 years, *P* = 0.009. Of 880 cases, 85.7% were farmers or herders, 94.3% had a history of animal exposure and 14.8% ingested unpasteurized foods. The subjects mainly presented with the acute stage, followed by subacute stage and chronic stage, accounting for 90.1%, 5.7% and 4.2%, respectively. Complications occurred more frequently in subacute group (*P* = 0.002). The proportion of complications is higher in the underlying disease group (*P* < 0.001). The median number of days from illness onset to definitive diagnosis was 22 days (*IQR*: 11–53) in complicated cases, and 15 days (*IQR*: 8–30) in uncomplicated cases (*P* < 0.001). There were no significant differences in the proportion of complications found among groups based on nationality, occupation, region, education, family history of brucellosis, or previous history of brucellosis (*P* > 0.05).

**Table 2:** Demographic and epidemiological characteristics of 880 brucellosis cases at the time of enrollment

Variables	Subgroups	Total *N* = 880 *n* (%)	Complicated *N* = 482 *n* (%)	Uncomplicated *N* = 398 *n* (%)	*P* value
Gender	Male	642 (73.0)	354 (73.4)	288 (72.4)	0.719
	Female	238 (27.0)	128 (26.6)	110 (27.6)	
Age group	< 60 years	702 (79.8)	369 (76.6)	333 (83.7)	0.009
	≥ 60 years	178 (20.2)	113 (23.4)	65 (16.3)	
Nationality	Han nationality	614 (69.8)	353 (73.3)	261 (65.6)	0.014
	Minority nationality	266 (30.2)	129 (26.7)	137 (34.4)	
Occupation	Farmer& Herdman	754 (85.7)	418 (86.7)	336 (84.4)	0.234
	Animal and animal products processing management	49 (5.6)	21 (4.4)	28 (7.0)	
	Other occupations^a^	77 (8.8)	43 (8.9)	34 (8.5)	
Region	Xinjiang	385 (43.8)	206 (42.7)	179 (45.0)	0.426
	Shandong	289 (32.8)	155 (32.2)	134 (33.7)	
	Inner Mongolia	206 (23.4)	121 (25.0)	85 (21.4)	
Education	Primary or below	364 (41.4)	216 (44.8)	148 (37.2)	0.051
	Junior high school	394 (44.8)	199 (41.3)	195 (49.0)	
	High school or above	122 (13.9)	67 (13.9)	55 (13.8)	
Exposure history^d^	Exposure to animals	830 (94.3)	442 (91.7)	388 (97.5)	< 0.001
	Sheep & goats	774 (88.0)	417 (86.5)	357 (89.7)	0.149
	Cattle	328 (37.3)	170 (35.3)	158 (39.7)	0.176
	Other animals^b^	22 5 (27.1)	145 (32.8)	80 (20.6)	< 0.001
Method of exposures to animals^d^	Raising	691 (78.5)	387 (80.3)	304 (76.4)	0.160
	Grazing	356 (40.5)	176 (36.5)	180 (45.2)	0.009
	Slaughter	222 (25.2)	132 (27.4)	90 (22.6)	0.105
	Delivering lambs	414 (47.1)	239 (49.6)	175 (44.0)	0.097
	Other routes^c^	369 (41.9)	195 (40.5)	174 (43.7)	0.329
Ingestion of unpasteurized food	Yes	130 (14.8)	97 (20.2)	33 (8.3)	< 0.001
	No	748 (85.2)	383 (79.8)	365 (91.7)	
Family history of brucellosis	Yes	183 (21.1)	96 (20.2)	87 (22.1)	0.489
	No	685 (78.9)	379 (79.8)	306 (77.9)	
Previous history of brucellosis	Yes	77 (8.8)	42 (8.7)	35 (8.8)	0.967
	No	803 (91.2)	440 (91.3)	363 (91.2)	
Days from onset to diagnosis	0–7	166 (18.9)	72 (14.9)	94 (23.8)	< 0.001
	8–30	425 (48.5)	219 (45.4)	206 (52.2)	
	31–90	207 (23.6)	132 (27.4)	75 (19.0)	
	> 90	79 (9.0)	59 (12.2)	20 (5.1)	
Clinical stage^e^	Acute	793 (90.1)	419 (86.9)	374 (94.0)	0.002
	Subacute	50 (5.7)	38 (7.9)	12 (3.0)	
	Chronic	37 (4.2)	25 (5.2)	12 (3.0)	
Underlying disease	Yes	311 (35.3)	198 (41.1)	113 (28.4)	< 0.001
	No	569 (64.7)	284 (58.9)	285 (71.6)	
Hospitalized	Yes	614 (71.8)	395 (84.6)	219 (56.4)	< 0.001
	No	241 (28.2)	72 (15.4)	169 (43.6)	

Those with missing values were excluded from analysis. Percentages may not sum to 100 due to rounding

^a^Other occupations include staff, student, teacher, and other non-occupational population with brucellosis

^b^Other animals include pigs, dogs, horses, deer, and camels

^c^Other routes include veterinarian, animal trade, animal product processing and sheep clipping

^d^Patients may have a history of multiple animal contacts or exposure modes. Therefore, the cumulative total may exceed 100%

^e^Inter-group comparison with the method of Bonferroni found that the proportion of complications among the three groups was different

### Clinical presentation and laboratory findings

The most prevalent symptoms among all patients were arthralgia (83.2%), fatigue (71.1%), fever (53.0%), sweating (49.8%), and inappetence (36.6%). The most commonly affected joints were the knee (37.3%), spine (34.5%), shoulder (21.9%), sacral (19.4%), and iliac (19.0%). Furthermore, elevated CRP levels (> 10 mg/L) were observed in 48.9% of patients, while elevated ESR was seen in 54.3% of patients.

The symptoms and signs observed in patients with focal brucellosis were similar to those without focal complications, except for a higher occurrence of arthralgia, inappetence, nausea, orchialgia, and eye bulging pain (*P* < 0.05). Patients with focal brucellosis were more likely to present with arthritis in large joints such as sacral, iliac, shoulder, and spine (*P* < 0.05). Fatigue was more common in patients without focal brucellosis (67.2% vs 75.9%, *P* < 0.05). The levels of ESR and CRP were higher in patients with complicated brucellosis compared to those with uncomplicated brucellosis (*P* < 0.001). Moreover, the rate of *Brucella* culture positivity was higher in patients with uncomplicated brucellosis compared to those with complicated brucellosis (6.9% vs 14.1%, *P* < 0.001). Please refer to Table 3 for additional information.

**Table 3:** Clinical characteristics and laboratory findings between patients with or without complications of human brucellosis

	Total *N* = 880 *n* (%)	Complicated *N* = 482 *n* (%)	Uncomplicated *N* = 398 *n* (%)	*P* value
Symptoms and signs				
Fever	466 (53.0)	256 (53.1)	210 (52.8)	0.918
Chills	193 (21.9)	104 (21.6)	89 (22.4)	0.779
Fatigue	626 (71.1)	324 (67.2)	302 (75.9)	0.005
Sweating	437 (49.7)	236 (49.0)	201 (50.5)	0.649
Arthralgia	732 (83.2)	438 (90.9)	294 (73.9)	< 0.001
Sacral	166 (19.4)	104 (21.9)	62 (16.3)	0.037
Iliac	162 (19.0)	109 (23.0)	53 (13.9)	0.001
Shoulder	187 (21.9)	117 (24.7)	70 (18.4)	0.027
Knee	319 (37.3)	181 (38.2)	138 (36.2)	0.555
Elbow	131 (15.3)	80 (16.9)	51 (13.4)	0.159
Wrist	93 (10.9)	60 (12.7)	33 (8.7)	0.062
Ankle	107 (12.5)	68 (14.4)	39 (10.2)	0.071
Spine	295 (34.5)	202 (42.6)	93 (24.4)	< 0.001
Myalgia	246 (28.0)	123 (25.5)	123 (30.9)	0.076
Cough	128 (14.6)	79 (16.4)	49 (12.3)	0.088
Inappetence	322 (36.6)	192 (39.8)	130 (32.7)	0.028
Nausea	94 (10.7)	63 (13.1)	31 (7.8)	0.012
Headache	254 (28.9)	143 (29.7)	111 (27.9)	0.562
Orchialgia	45 (5.1)	40 (8.3)	5 (1.3)	< 0.001
Weight loss	273 (31.0)	162 (33.6)	111 (27.9)	0.068
Sleep disturbance	82 (9.3)	52 (10.8)	30 (7.5)	0.099
Vomit	35 (4.0)	24 (5.0)	11 (2.8)	0.094
Eye bulging pain	38 (4.3)	30 (6.2)	8 (2.0)	0.002
Urgent micturition and frequent micturition	41 (4.7)	27 (5.6)	14 (3.5)	0.144
Rashes	3 (0.3)	2 (0.4)	1 (0.3)	1.000
Hematologic				
Anemia	121 (14.1)	77 (16.4)	44 (11.3)	0.033
Leukopenia	111 (12.9)	65 (13.8)	46 (11.8)	0.383
Leukocytosis	48 (5.6)	30 (6.4)	18 (4.6)	0.265
Thrombocytopenia	29 (3.4)	15 (3.2)	14 (3.6)	0.747
Serum biochemistry				
ALT > 40 U/L	303 (34.9)	173 (36.5)	130 (33.0)	0.281
AST > 42 U/L	200 (23.1)	109 (23.0)	91 (23.3)	0.923
Bilirubin > 18.6 μmol/L	148 (17.2)	88 (18.6)	60 (15.4)	0.207
Urea nitrogen > 7.14 mmol/L	86 (10.1)	51 (10.9)	35 (9.0)	0.356
Creatinine > 124 μmol/L	2 (0.2)	2 (0.4)	0	0.504
Inflammatory markers				
CRP > 10 mg/L	399 (48.9)	258 (56.6)	141 (39.2)	< 0.001
ESR elevation	415 (54.3)	264 (62.0)	151 (44.5)	< 0.001
Bacterial culture and serum-antibody-test				
Culture positive	89 (10.1)	33 (6.9)	56 (14.1)	< 0.001
SAT ≥ 200	666 (77.0)	359 (76.1)	307 (78.1)	0.474
SAT ≥ 400	387 (44.7)	193 (40.9)	194 (49.4)	0.013

*ALT* Alanine aminotransferase, *AST* Aspartate aminotransferase, *CRP* C-reactive protein, *ESR* Erythrocyte sedimentation rate, *SAT* Standard tube agglutination test

### Warning signs associated with complications

The univariate logistic regression analysis revealed that several factors were significantly associated with focal brucellosis, including age, nationality, time from onset to diagnosis, presence of underlying disease, fatigue, arthralgia, inappetence, nausea, orchialgia, eye bulging pain, anemia, CRP > 10 mg/L, ESR elevation, positive culture, and SAT ≥ 400.

**Fig. 1:**
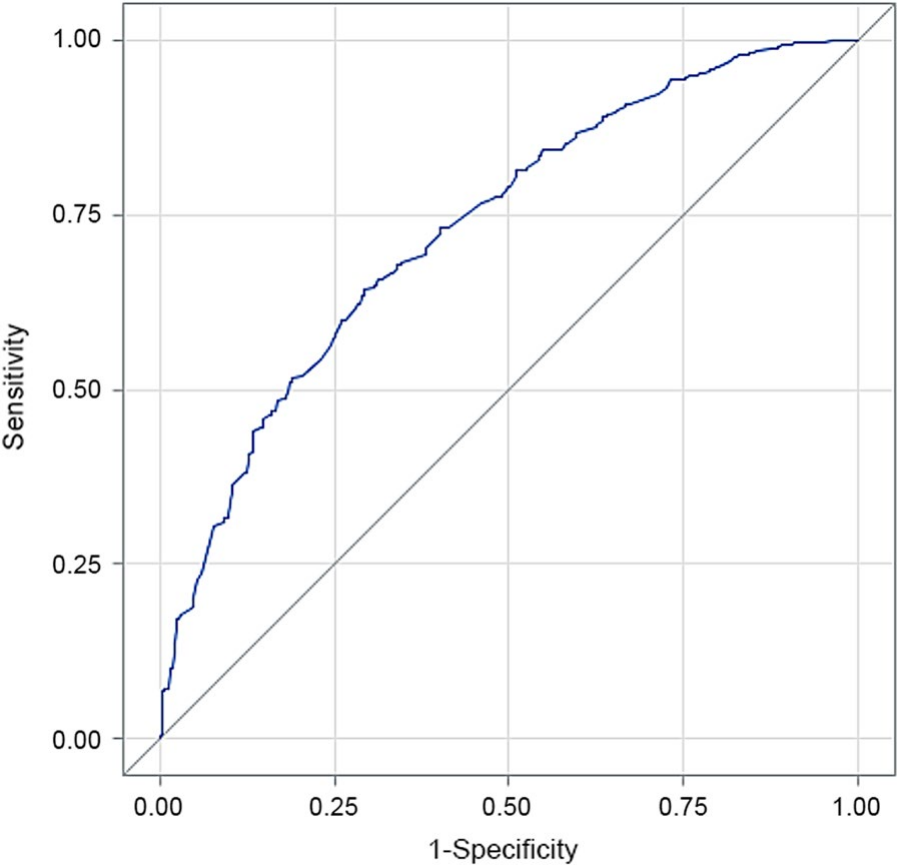
ROC of multivariate logistic regression analysis model of warning signs of human brucellosis complications

We conducted multivariate logistic regression analysis to identify independent factors associated with complications. Our findings indicate that several factors were independently associated with focal brucellosis, including the number of days from onset to diagnosis, underlying disease, symptoms such as arthralgia and myalgia, eye bulging pain, CRP > 10 mg/L, ESR elevation, positive culture, and SAT ≥ 400 (Table 4). The model demonstrated good prediction performance with an AUC of 0.732, as shown in Fig. 1.

**Table 4:** Univariate and multivariate logistic regression analysis of warning signs of human brucellosis complications

Variables	Univariate analysis				Multivariate analysis^a^			
	Wald *χ*^*2*^	*OR*	*OR* (95% *CIs*)	*P* value	Wald *χ*^*2*^	*OR*	*OR* (95% *CIs*)	*P* value
Days from onset to diagnosis	29.142	-	-	< 0.001	19.418	-	-	< 0.001
0–7	-	-	1 (reference)	-	-	-	1 (reference)	-
8–30	3.166	1.388	0.967–1.992	0.075	4.282	1.610	1.025–2.527	0.039
31–90	15.233	2.298	1.513–3.489	< 0.001	12.217	2.551	1.509–4.314	0.001
> 90	10.877	3.851	2.129–6.967	< 0.001	13.601	3.963	1.906–8.238	< 0.001
Underlying disease	15.219	1.758	1.324–2.335	< 0.001	8.180	1.675	1.176–2.384	0.004
Arthralgia	41.655	3.520	2.402–5.159	< 0.001	22.869	3.197	1.986–5.148	< 0.001
Myalgia	3.132	0.766	0.570–1.029	0.077	5.914	0.627	0.431–0.913	0.015
Eye bulging pain	8.453	3.236	1.466–7.14	0.004	6.649	3.482	1.349–8.988	0.001
CRP > 10 mg/L	24.144	2.024	1.528–2.681	< 0.001	11.316	1.910	1.310–2.784	0.001
ESR elevation	22.854	2.029	1.518–2.712	< 0.001	7.147	1.663	1.145–2.415	0.008
Culture positive	12.037	0.449	0.285–0.706	0.001	5.060	0.516	0.290–0.918	0.025
SAT ≥ 400	6.213	0.710	0.542–0.929	0.013	13.209	0.529	0.376–0.746	< 0.001
Age < 60 years	6.772	0.637	0.454–0.895	0.009	-	-	-	-
Nationality, Han	6.039	1.436	1.076–1.917	0.014	-	-	-	-
Education	5.959	-	-	0.051				
Primary or below	-	-	1 (reference)	-	-	-	-	-
Junior high school	5.942	0.699	0.524–0.932	0.015	-	-	-	-
High school or above	0.975	0.806	0.525–1.237	0.324	-	-	-	-
Fatigue	7.912	0.652	0.484–0.878	0.005	-	-	-	-
Inappetence	4.819	1.365	1.034–1.802	0.028	-	-	-	-
Nausea	6.244	1.780	1.132–2.798	0.013	-	-	-	-
Orchialgia	16.750	7.113	2.780–18.202	< 0.001	-	-	-	-
Anemia	4.479	1.536	1.032–2.286	0.034	-	-	-	-
Weight loss	0.068	1.309	0.980–1.748	0.068	-	-	-	-

*CRP* C-reactive protein, *ESR* Erythrocyte sedimentation rate, *SAT* Standard tube agglutination test, - Not appliable. ^a^The final model was established through model evaluation and model diagnosis. Variables without statistical significance were excluded

### ROC curve analysis of clinical biomarkers in patients with and without complications

**Fig. 2:**
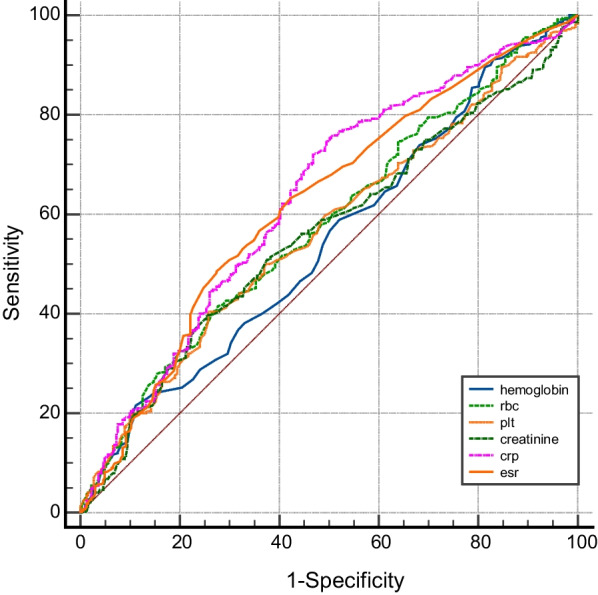
ROC analyses for various cutoff values of laboratory parameters in predicting complications associated with human brucellosis

The AUC values for RBC, Hb, PLT, creatinine, CRP, and ESR as indicators of complicated cases were 0.576 (95% *CI* 0.542–0.610, *P* < 0.001), 0.546 (95% *CI* 0.512–0.580, *P* = 0.019), 0.546 (95% *CI* 0.512–0.580, *P* = 0.019), 0.567 (95% *CI* 0.533–0.600, *P* = 0.001), 0.634 (95% *CI* 0.598–0.668, *P* < 0.001), and 0.607 (95% *CI* 0.571–0.642, *P* < 0.001) (Additional file 1: Table S1). The optimal cutoff value determined by the ROC analysis was > 5.4 mg/L for CRP (sensitivity 73.4% and specificity 51.9%) and > 25 mm/h for ESR (sensitivity 47.9% and specificity 71.1%) (Fig. 2).

## Discussion

Our study aimed to compare the demographic, clinical, and laboratory characteristics of brucellosis patients with or without complications to provide practical reference indices for clinicians in early diagnosis and patient management. More than half of the brucellosis patients experienced complications. Several factors were identified as warning signs for complications, including delayed diagnosis, underlying diseases, arthralgia, myalgia, eye bulging pain, CRP levels greater than 10 mg/L, and elevated ESR levels.

Large-scale epidemiological studies have reported complication rates ranging from 27.7 to 90% [11, 14]. The main affected anatomical sites were the osteoarticular, hematologic, and genitourinary systems, with incidence ranging from 2 to 77%, 2% to 53%, and 2% to 20%, respectively [15]. In this study, complications were observed in 54.8% of brucellosis cases, with osteoarticular involvement being the most common, consistent with previous studies. Therefore, brucellosis should be considered in the differential diagnosis of osteoarthropathy, orchitis, and other physiotherapy-related diseases in the rehabilitation department, especially in endemic regions. The spectrum of clinical symptoms and the distribution of focal involvement were found to be different from previous studies [16–18]. In our study, the complicated group showed a significantly higher incidence of arthralgia, inappetence, nausea, orchialgia, and eye bulging pain, while fatigue was significantly less frequent compared to the uncomplicated group. These differences may be attributed to variations in the definition of complications, diagnostic methods and instruments, epidemiological research methods, and the characteristics of the study subjects. For instance, a large epidemiological study conducted in China among inpatients at a high-level hospital reported a high incidence of complications at 90%, with 26% classified as subacute or chronic type [11]. The subjects of our research may be considered representative of brucellosis patients in China due to the proper study design, thus providing a comprehensive profile of the clinical spectrum of complicated brucellosis in the country.

In our study, we found that arthralgia, loss of myalgia, and eye bulging pain were statistically significant indicators of complications in patients. Specifically, osteoarticular complications were more commonly seen in large joints, such as the spine. Our results align with previous studies, which also identified arthralgia, absence of myalgias, and low back pain as associated factors for complications [16, 19–21]. Notably, eye bulging pain, although not common in brucellosis patients, should be carefully considered during diagnosis and treatment.

Several previous studies have shown that delayed diagnosis of brucellosis can lead to an increased rate of complications [19, 22, 23]. The longer brucellosis symptoms persist, the higher the risk of complications, likely due to the prolonged presence of the bacteria in the body. Therefore, early diagnosis is crucial in order to prevent complications and achieve better clinical outcomes. However, delayed diagnosis is a common occurrence, with medical practitioners citing the various atypical clinical manifestations of brucellosis and its resemblance to other diseases as major factors [24]. Additionally, prompt and accurate identification of complications is important, as extended treatment and the use of additional antibiotics are necessary for complete eradication of *Brucella* in patients with focal brucellosis [10]. Inadequate and inappropriate treatment can easily lead to chronicity [25], resulting in significant harm to patients and imposing a heavy economic burden on both their families and society as a whole. Therefore, it is essential to explore the use of biomarkers as comprehensive diagnostic indicators for complicated brucellosis.

This study identified CRP and ESR as indicators of focal involvement in brucellosis, with a cutoff value of 5.4 mg/L for CRP and 25 mm/h for ESR. Consistent with these findings, other studies have also reported higher levels of CRP and ESR in patients with complications [19]. Betul et al. [21] discovered that an ESR > 30 mm/h was a predictive factor for brucellosis complications. Additionally, Colmenero et al. [22] found that the risk of organ involvement was roughly doubled in cases of brucellosis diagnosed 30 days later with an ESR > 40 mm/h. Furthermore, the titer levels of IgG, IgM, and the neutrophil–lymphocyte ratio in the serum have been associated with complications [12, 26, 27].

The utility of blood culture for early identification of complications in brucellosis remains controversial, with limited and conflicting evidence available. Xu et al. [12] found that positive blood cultures were indicative of complicated brucellosis, while Bircan et al. [19] reported that negative blood cultures were statistically significant factors for complications. In other studies [20–22], the rates of blood culture and SAT positivity were similar in groups with and without focal involvement. The accuracy of blood culture results was greatly influenced by the conditions and techniques used [8]. In our study, we observed that negative blood cultures and low SAT titers were associated with brucellosis complications, suggesting the need for further in-depth research on this topic.

This study has several limitations. First, we selected provinces with a high number of brucellosis cases as study sites, which limited our ability to explore the association between *Brucella* strain type and brucellosis complications. Second, due to financial constraints, we were unable to include certain biomarkers related to immunity, such as CD4+ T lymphocyte, CD8+ T lymphocyte, IL2, and IL6. Third, using conventional indicators like inflammatory markers may not be appropriate as they can be influenced by autoimmune conditions and immunodeficiency diseases. Furthermore, future research should incorporate metabolomics, proteomics, and immunomics to detect biomarkers of brucellosis complications in serum and interstitial fluid. Such expansion of research is warranted in the future.

## Conclusions

Complications are a frequent occurrence in patients with brucellosis. It is important to closely monitor patients with delayed diagnosis, underlying diseases, significant joint pain, eye bulging pain, and elevated levels of CRP or ESR during their initial visit. Additionally, early treatment should be administered with vigilance to prevent the onset of complications.

### Supplementary Information

**Additional file 1: Table S1.** ROC analyses for various cutoff values of laboratory parameters in predicting complications associated with human brucellosis.

## Data Availability

The datasets produced and/or analyzed in this study can be obtained from the corresponding author upon request.

## Abbreviations

ALT: Alanine aminotransferase
AST: Aspartate aminotransferase
AUC: Area under the curve
CI: Confidence intervals
CRP: C-reactive protein
CT: Computed tomography
ECG: Electrocardiogram
ESR: Erythrocyte sedimentation rate
Hb: Hemoglobin
IQR: Interquartile range
MRI: Magnetic resonance imaging
*OR*: Odds ratio
PLT: Platelets
RBC: Red blood cells
ROC: Receiver operating characteristic
SAT: Standard tube agglutination test
UCG: Ultrasound cardiogram
WBC: White blood cells
