# Supplementary material for: Incidence and warning signs for complications of human brucellosis: a multi-center observational study from China
Source: Infect Dis Poverty. 2024 Feb 20;13:18. doi: 10.1186/s40249-024-01186-4 (PMC10877768; doi:10.1186/s40249-024-01186-4)
Supplement: Supplementary file 1 — Additional file 1: Table S1. ROC analyses for various cutoff values of laboratory parameters in predicting complications associated with human brucellosis. [file 40249_2024_1186_MOESM1_ESM.docx]

**Table S1**. ROC analyses for various cutoff values of laboratory parameters in predicting complications associated with human brucellosis.

| **Warning factors** | **AUC** | **95%*CI*** | ***P* value** | **Cut-Off** | **Sensitivity** | **Specificity** |
| --- | --- | --- | --- | --- | --- | --- |
| RBC | 0.576 | 0.542–0.610 | < 0.001 | 4.3 | 41.2 | 72.0 |
| Hb | 0.546 | 0.512–0.580 | 0.019 | 117.0 | 20.4 | 87.9 |
| WBC | 0.502 | 0.468–0.536 | 0.938 | 3.7 | 11.9 | 93.3 |
| PLT | 0.546 | 0.512–0.580 | 0.019 | 255.0 | 38.4 | 73.0 |
| ALT | 0.507 | 0.473–0.540 | 0.740 | 20.1 | 30.8 | 77.2 |
| AST | 0.514 | 0.480–0.548 | 0.481 | 17.0 | 21.5 | 87.0 |
| Bilirubin | 0.523 | 0.489–0.557 | 0.247 | 14.0 | 33.1 | 75.1 |
| Urea nitrogen | 0.532 | 0.498–0.566 | 0.103 | 5.8 | 30.7 | 78.0 |
| Creatinine | 0.567 | 0.533–0.600 | 0.001 | 60.7 | 44.2 | 72.4 |
| CRP | 0.634 | 0.598–0.668 | < 0.001 | 5.4 | 73.4 | 51.9 |
| ESR | 0.607 | 0.571–0.642 | < 0.001 | 25.0 | 47.9 | 71.1 |

ALT: Alanine aminotransferase, AST: Aspartate aminotransferase, CRP: C-reactive protein, ESR: Erythrocyte sedimentation rate, Hb: Hemoglobin, PLT: Platelets, RBC: Red blood cells, WBC: White blood cells.
